# iE-DAP Induced Inflammatory Response and Tight Junction Disruption in Bovine Mammary Epithelial Cells via NOD1-Dependent NF-κB and MLCK Signaling Pathway

**DOI:** 10.3390/ijms24076263

**Published:** 2023-03-27

**Authors:** Yan Wang, Xuerui Li, Zhengqiang Han, Meijuan Meng, Xiaoli Shi, Lairong Wang, Mengru Chen, Guangjun Chang, Xiangzhen Shen

**Affiliations:** 1 Ministry of Education Joint International Research Laboratory of Animal Health and Food Safety, College of Veterinary Medicine, Nanjing Agricultural University, Nanjing 210095, China; 2 Jinling Institute of Technology, Nanjing 210038, China; 3 College of Animal Science and Technology, Nanjing Agricultural University, Nanjing 210095, China

**Keywords:** bovine mammary epithelial cell, iE-DAP, inflammatory response, tight junction, NF-κB

## Abstract

*γ*-D-glutamyl-*meso*-diaminopimelic acid (iE-DAP), a bacterial cell wall component, can trigger an inflammatory response. A mammary inflammatory response causes tight junction (TJ) dysfunction. This study aimed to explore the effects and involved mechanisms of iE-DAP-induced inflammatory response on the TJ integrity in bovine mammary epithelial cells (BMECs). The results showed that iE-DAP-induced inflammatory response and TJ disruption was associated with increased expression levels of inflammatory cytokines and decreased gene expression of ZO-1 and Occludin, as well as a reduction in transepithelial electrical resistance and elevation in paracellular dextran passage. While MLCK inhibitor ML-7 reversed the TJ disruption induced by iE-DAP. NF-κB inhibitor BAY 11-7085 hindered the activation of NF-κB and MLCK signaling pathways, the inflammatory response and TJ disruption induced by iE-DAP. NOD1-specific shRNA also inhibited the activation of the NOD1/NF-κB signaling pathway and reversed the inflammatory response and TJ injury in iE-DAP-treated BMECs. Above results suggest that iE-DAP activated the NF-κB and MLCK signaling pathway in NOD1-dependent manner, which promoted the transcription of inflammatory cytokines and altered the expression and distribution of tight junction proteins, finally caused inflammatory response and TJ disruption. This study might provide theoretical basis and scientific support for the prevention and treatment of mastitis.

## 1. Introduction

In the modern dairy industry, mastitis, namely inflammation in the mammary gland, is a prevalent clinical disease. The occurrence of mastitis leads to the decline of milk production and milk quality, increase in the proportion of inferior milk and treatment expenditure, and finally causes enormous economic losses in the dairy industry [1,2]. At the same time, it also causes the destruction of mammary gland structure, endangers animal health and results in serious animal welfare issues [3]. Therefore, elucidating the molecular mechanism of mastitis is of great significance to the sustainable development of the dairy industry. Both stimulation of internal and external factors, including mechanical injury, pathogenic microbial infection, physical and chemical stimulation, can lead to mastitis [4]. In addition, previous studies have also shown that a high concentrate diet increased the concentration of bacterial endotoxins such as lipopolysaccharide (LPS) [5] and *γ*-D-glutamyl-*meso*-diaminopimelic acid (iE-DAP) [6], which induced the mammary inflammatory response.

iE-DAP, a dipeptide component of the peptidoglycan of Gram-negative bacteria and some Gram-positive bacteria, is the core structure recognized by the intracellular molecular pattern recognition receptor (PRR) nucleotide oligomerization binding domain protein 1 (NOD1) [7,8,9]. After recognizing and binding to iE-DAP, NOD1 can activate the NF-κB signaling pathway, which promotes the transcription of inflammation-related genes and induces an inflammatory response [10,11]. A previous study has shown that iE-DAP treatment could activate the NF-κB signaling pathway, which promoted the gene expression of inflammatory cytokines and chemokines and caused an inflammatory response in bovine hepatocytes [12]. However, the studies on the effects of iE-DAP-induced inflammatory injury on bovine mammary epithelial cells are still limited.

Bovine mammary epithelial cells (BMECs), as the most dominant cells in the mammary gland, can not only secrete milk protein but also play a critical role in the immune response of the mammary gland [13,14]. BMECs can recognize pathogenic microbials and regulate immune cells by secreting cytokines to promote their immune defense function [15]. Stimulation with bacterial endotoxins such as *Escherichia coli* or *Staphylococcus aureus* induced different degrees of inflammatory responses in BMECs [15]. At same time, BMECs also participate in the composition of the blood-milk barrier, which is an important physical barrier in mammary gland. A normal blood-milk barrier is essential for maintaining mammary health and physiological function [16,17]. A previous study has shown that mastitis caused by bacterial infection leads to pathogen-dependent damage to the blood-milk barrier accompanied by an increase in tight junction (TJ) permeability in BMECs [18]. In normal mammary gland tissue, TJs in BMECs are an important component of the blood-milk barrier, which can prevent milk components from leaking into plasma from the acinar cavity, and vice versa [19,20]. Transmembrane proteins including ZO-1, Occludin, Claudins and their complex combinations constitute TJs. The occurrence of mastitis will destroy the integrity of TJs, increase their permeability and damage mammary health [21]. A previous study has shown that an LPS-induced mammary inflammatory response altered the expression and distribution of TJ proteins and disrupted the milk-blood barrier [19]. Other studies have shown that excessive cytokines release, such as TNF-α, IL-1β, IL-6 and IL-8 could induce inflammation-related TJ destruction. On the contrary, the reduction of cytokine concentration facilitated the recovery of TJ integrity [22,23,24]. However, the effects and mechanism of iE-DAP-induced inflammatory response on TJs in BMECs are rarely known.

Therefore, in order to investigate the effects and involved mechanism of iE-DAP-induced inflammatory response on TJ integrity and barrier function in BMECs, the expression and secretion of inflammatory cytokines, the expression and distribution of TJ proteins, the permeability of TJs and the activation of the inflammatory signaling pathway in BMECs after iE-DAP treatment were detected. The results were further verified by using inhibitors and short hairpin RNA (shRNA). We hypothesized that iE-DAP-induced inflammatory response might lead to the changes in the expression and distribution of TJ proteins and destroy the integrity of TJs in BMECs through activating the NOD1/NF-κB signaling pathway.

## 2. Results

### 2.1. Time Gradient Effects of iE-DAP Treatment on the mRNA Expression of Inflammatory Cytokines and Tight Junction Protein Genes in BMECs

**Figure 1:**
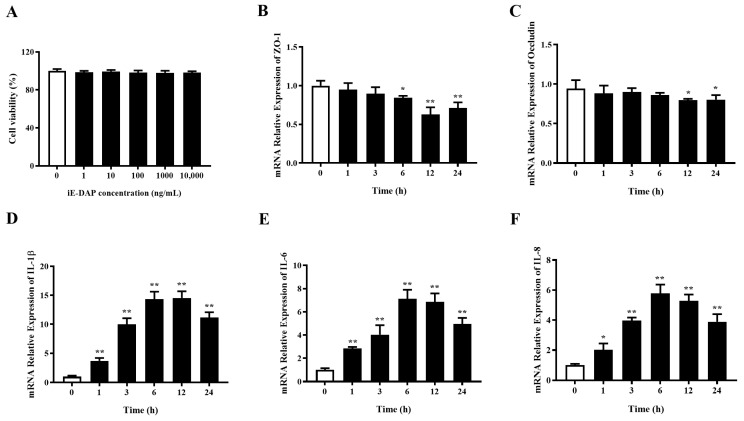
The mRNA expression of inflammatory cytokines and tight junction (TJ) protein genes in bovine mammary epithelial cells (BMECs) after *γ*-D-glutamyl-*meso*-diaminopimelic acid (iE-DAP) treatment at different time points. (**A**) Cell viability of BMECs treated with different concentration of iE-DAP. (**B**,**C**) Gene expression of TJ proteins and (**D**–**F**) inflammatory cytokines in BMECs treated with 1000 ng/mL iE-DAP at different time points (0, 1, 3, 6, 12 and 24 h). Results were expressed as the mean ± SEM. * *p* < 0.05, ** *p* < 0.01, representing significant difference compared with the control group.

Figure 1A shows that treatment with different concentration of iE-DAP (1, 10, 100, 1000 and 10,000 ng/mL) had no cytotoxic effect on the BMECs. The time gradient test of iE-DAP treatment was carried out to optimize the treatment time according to the changes in the relative mRNA expression of inflammatory cytokines and TJ proteins. As can be seen from Figure 1D–F, the mRNA relative expression of inflammatory cytokines IL-1β (*p* < 0.01), IL-6 (*p* < 0.01) and IL-8 (*p* < 0.05) were significantly upregulated after iE-DAP treatment for 1 h compared with the control group, which increased in a time-dependent manner. Figure 1B,C shows that iE-DAP treatment for 6 h significantly downregulated the mRNA relative expression of ZO-1 (*p* < 0.05) in comparison to the control group. While after iE-DAP treatment for 12 h, the mRNA relative expression of Occludin was significantly decreased (*p* < 0.05) compared to the control group. The gene expression of TJ proteins was also downregulated in a time-dependent manner. Considering the changes in gene expression of inflammatory cytokines and TJ proteins, 12 h was finally selected as the subsequent treatment time of iE-DAP.

### 2.2. Gradient Effects of iE-DAP Treatment on the Inflammatory Response and Tight Junction Integrity in BMECs

**Figure 2:**
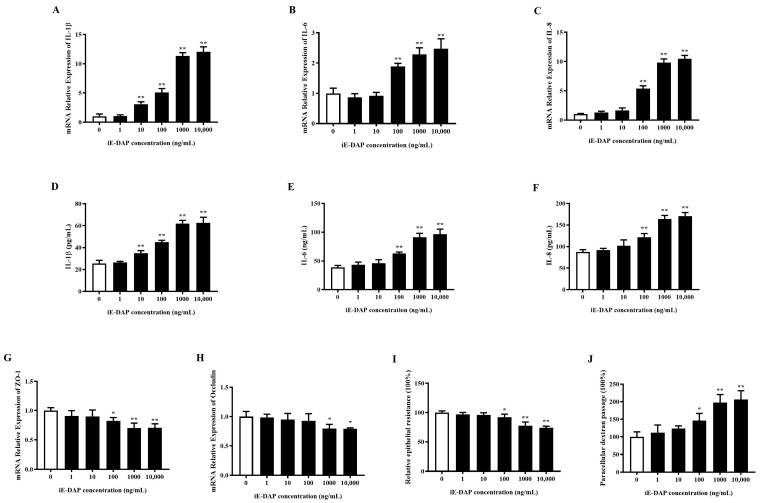
Effects of iE-DAP treatment on the inflammatory response and TJ integrity in BMECs. (**A**–**C**) Gene expression of inflammatory cytokines in BMECs after treatment with 0, 1, 10, 100, 1000 and 10,000 ng/mL iE-DAP for 12 h. (**D**–**F**) The concentration of inflammatory cytokines in culture supernatant of BMECs after treatment with iE-DAP. (**G**,**H**) Gene expression of TJ proteins in BMECs treated with different concentration iE-DAP for 12 h. (**I**) Relative epithelial resistance and (**J**) paracellular dextran passage of the BMEC monolayer after iE-DAP treatment. Results are shown as the mean ± SEM. * *p* < 0.05, ** *p* < 0.01, representing significant differences compared with the control group.

As shown in Figure 2A–F, 10 ng/mL of iE-DAP significantly increased the mRNA relative expression and release of IL-1β (*p* < 0.01). The expression levels of IL-6 and IL-8 (*p* < 0.01) were significantly increased with 100 ng/mL iE-DAP treatment. Additionally, iE-DAP increased the gene expression and release of inflammatory cytokines in a concentration-dependent manner. According to the RT-qPCR results, 100 ng/mL iE-DAP significantly reduced the relative mRNA expression of ZO-1 (*p* < 0.05), and 1000 ng/mL iE-DAP significantly reduced that of Occludin (*p* < 0.05). iE-DAP treatment decreased the gene expression of TJ proteins in a concentration-dependent manner (Figure 2G,H). Transepithelial electrical resistance (TEER) and paracellular permeability were conducted to further evaluate the effects of iE-DAP on the integrity of BMEC monolayers. As Figure 2I,J shows, 100 ng/mL iE-DAP significantly decreased the TEER (*p* < 0.05) and increased paracellular dextran passage (*p* < 0.05) compared to the control group.

### 2.3. Effects of ML-7 Pretreatment on Tight Junction Disruption in BMECs Induced by iE-DAP

**Figure 3:**
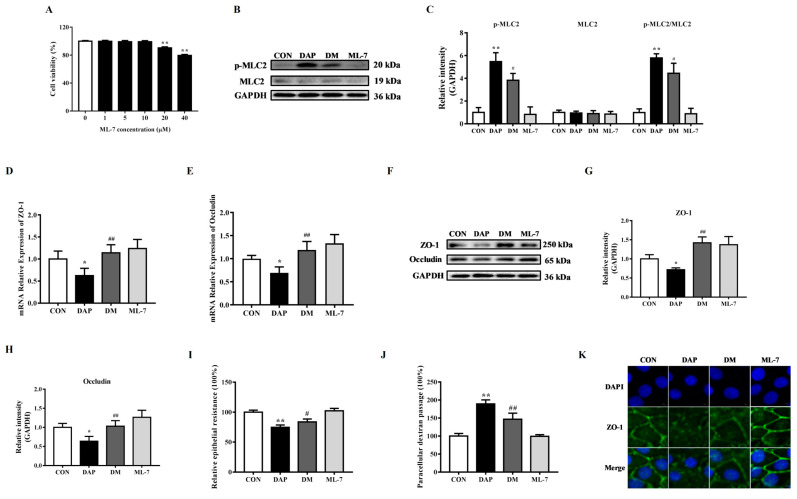
Effects of ML-7 on the TJ disruption induced by iE-DAP treatment in BMECs. (**A**) Cell viability of BMECs treated with different concentration of ML-7. (**B**,**C**) WB representative bands and quantitative results of MLC2 and p-MLC2 proteins in BMECs treated with 1000 ng/mL iE-DAP for 12 h after 10 μM ML-7 pretreatment for 2 h. (**D**,**E**) Gene expression of ZO-1 and OCCLUDIN and (**F**–**H**) WB representative bands and quantitative results of ZO-1 and Occludin in BMECs treated with iE-DAP after ML-7 pretreatment. (**I**) Relative epithelial resistance and (**J**) paracellular dextran passage of the BMEC monolayer treated with iE-DAP after ML-7 pretreatment. (**K**) Immunofluorescence results of ZO-1 in BMECs treated with iE-DAP after ML-7 pretreatment. In the immunofluorescence results, the scale bar = 5 μm. Results were expressed as the mean ± SEM. * *p* < 0.05, ** *p* < 0.01, represent a significant difference compared with the control group. # *p* < 0.05, ## *p* < 0.01, represent a significant difference compared with iE-DAP group. CON: control group; DAP: iE-DAP group; DM: ML-7 + iE-DAP group; ML-7: ML-7 group.

MLCK has been found to be involved in the regulation of TJ proteins [25]. In order to verify the role of MLCK in the TJ disruption in iE-DAP-treated BMECs, MLCK inhibitor ML-7 was selected. First of all, CCK-8 results demonstrated that 1~10 μM ML-7 had no significant effect on the cell viability. So that 10 μM ML-7 was employed in a subsequent experiment (Figure 3A). Figure 3B,C displayed that iE-DAP treatment significantly increased the protein expression of phosphorylated MLC2 (*p* < 0.01) and the phosphorylation ratio of MLC2 (*p* < 0.01). However, ML-7 pretreatment significantly reduced the protein level of phosphorylated MLC2 (*p* < 0.05) and the phosphorylation ratio of MLC2 (*p* < 0.05) compared with the iE-DAP group. Figure 3D,E shows that 10 μM ML-7 pretreatment for 2 h could inhibit the downregulation in the gene expression of ZO-1 (*p* < 0.01) and Occludin (*p* < 0.01) induced by iE-DAP. WB results also showed the same trend as gene expression (Figure 3F–H). The results of cellular immunofluorescence further verified that inhibition of MLCK could reverse the reduction in the protein expression and redistribution of ZO-1 induced by iE-DAP (Figure 3K). Figure 3I,J shows that ML-7 blocked the decrease in the TEER (*p* < 0.05) and increase in paracellular dextran passage (*p* < 0.01).

### 2.4. Effects of BAY 11-7085 Pretreatment on the Inflammatory Response in BMECs Induced by iE-DAP

**Figure 4:**
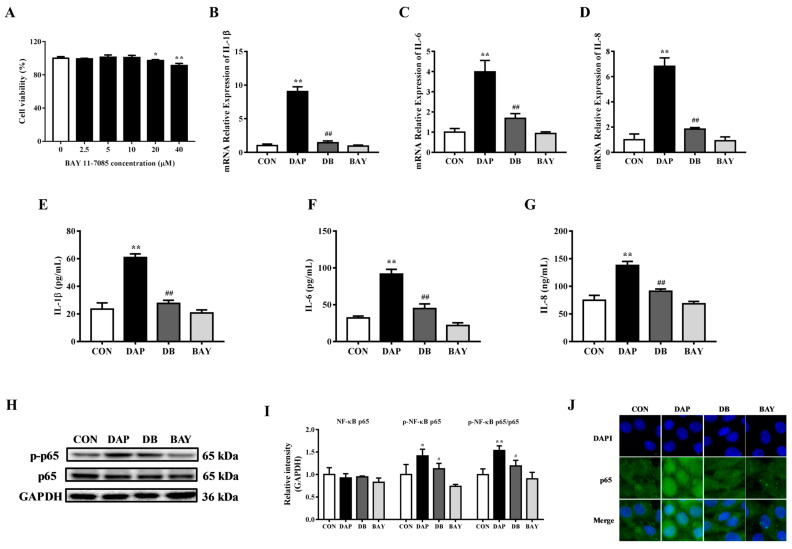
Effects of BAY 11-7085 on the inflammatory response induced by iE-DAP in BMECs. (**A**) Cell viability of BMECs treated with different concentration of BAY 11-7085. (**B**–**D**) Gene expression of inflammatory cytokines in BMECs treated with 1000 ng/mL iE-DAP for 12 h after 10 μM BAY 11-7085 pretreatment for 1 h. (**E**–**G**) The concentration of inflammatory cytokines in culture supernatant of BMECs treated with iE-DAP after BAY 11-7085 pretreatment. (**H**,**I**) WB representative bands and quantitative results of NF-κB signaling pathway-related proteins and (**J**) immunofluorescence results of NF-κB p65 in BMECs treated with iE-DAP after BAY 11-7085 pretreatment. In the immunofluorescence results, the scale bar = 5 μm. Results were shown as the mean ± SEM. * *p* < 0.05, ** *p* < 0.01, represent a significant difference compared with the control group. # *p* < 0.05, ## *p* < 0.01, represent a significant difference compared with the iE-DAP group. DB: BAY 11-7085 + iE-DAP group; BAY: BAY 11-7085 group.

As an important transcription factor, NF-κB participates in the regulation of inflammatory responses [26]. To study the influence of NF-κB in the inflammatory response of BMECs induced by iE-DAP, BAY 11-7085 was selected to inhibit the activity of NF-κB. In comparison to the control group, 20 and 40 μM BAY 11-7085 (*p* < 0.01) significantly decreased the cell viability of BMECs, while 1~10 μM exerted no significant cytotoxic effect (Figure 4A). It can be seen from Figure 4B–G, 10 μM BAY 11-7085 pretreatment for 1 h could hinder the increase in the transcription and release of the inflammatory cytokines IL-1β (*p* < 0.01), IL-6 (*p* < 0.01) and IL-8 (*p* < 0.01) induced by iE-DAP stimulation. WB results further showed that BAY 11-7085 pretreatment significantly decreased the protein abundance of phosphorylated NF-κB p65 (*p* < 0.05) and the phosphorylation ratio of NF-κB p65 (*p* < 0.01) compared with the iE-DAP group (Figure 4H,I). Meanwhile, immunofluorescence results further showed that BAY 11-7085 pretreatment also reduced the NF-κB p65 fluorescence expression in the nucleus caused by iE-DAP (Figure 4J).

### 2.5. Effects of BAY 11-7085 Pretreatment on the Tight Junction Disruption in BMECs Induced by iE-DAP

**Figure 5:**
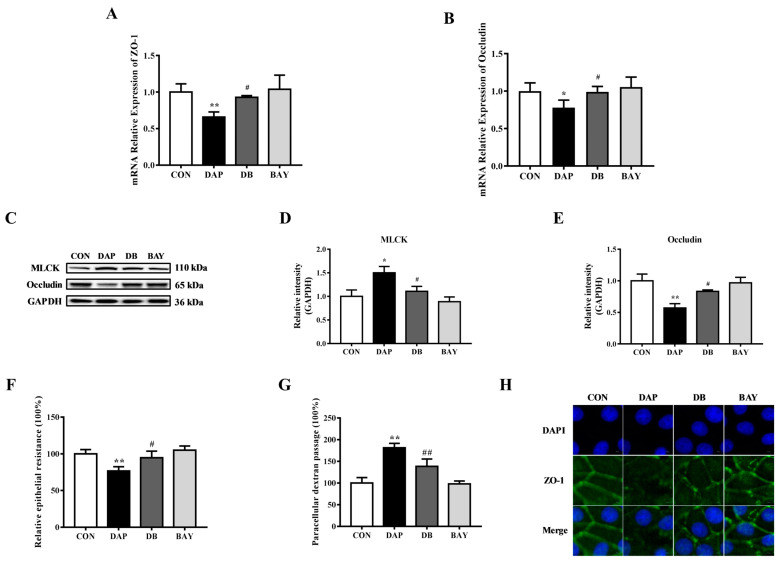
Effects of BAY 11-7085 on the TJ disruption induced by iE-DAP in BMECs. (**A**,**B**) Gene expression of ZO-1 and Occludin in BMECs treated with 1000 ng/mL iE-DAP for 12 h after 10 μM BAY 11-7085 pretreatment for 1 h. (**C**–**E**) WB representative bands and quantitative results of MLCK and Occludin in BMECs treated with iE-DAP after BAY 11-7085 pretreatment. (**F**) Relative epithelial resistance and (**G**) paracellular dextran passage of the BMEC monolayer treated with iE-DAP after BAY 11-7085 pretreatment. (**H**) Immunofluorescence results of ZO-1 in BMECs treated with iE-DAP after BAY 11-7085 pretreatment. In the immunofluorescence results, the scale bar = 5 μm. Results were presented as the mean ± SEM. * *p* < 0.05, ** *p* < 0.01, represent a significant difference compared with the control group. # *p* < 0.05, ## *p* < 0.01, represent a significant difference compared with iE-DAP group.

To further explore the role of NF-κB on the TJ disruption in BMECs induced by iE-DAP, the effects of BAY 11-7085 pretreatment on the expression, distribution and function of TJ proteins were examined. RT-qPCR results showed that BAY 11-7085 pretreatment reversed the downregulation of gene expression of ZO-1 (*p* < 0.05) and Occludin (*p* < 0.05) induced by iE-DAP stimulation (Figure 5A,B). WB results further verified that the protein level of Occludin demonstrated the same trend as gene expression (Figure 5C,E). Meanwhile, BAY 11-7085 pretreatment reversed the increase in the protein expression of MLCK (*p* < 0.05) caused by iE-DAP stimulation (Figure 5C,D). The results of cellular immunofluorescence also showed that BAY 11-7085 pretreatment reversed decreased fluorescence expression and redistribution of ZO-1 caused by iE-DAP stimulation (Figure 5H). As shown in Figure 5F,G, BAY 11-7085 also improved the TJ integrity with an elevation in the TEER (*p* < 0.05) and a reduction in paracellular dextran passage (*p* < 0.01).

### 2.6. Effects of NOD1 Interference on the Inflammatory Response in BMECs Induced by iE-DAP

**Figure 6:**
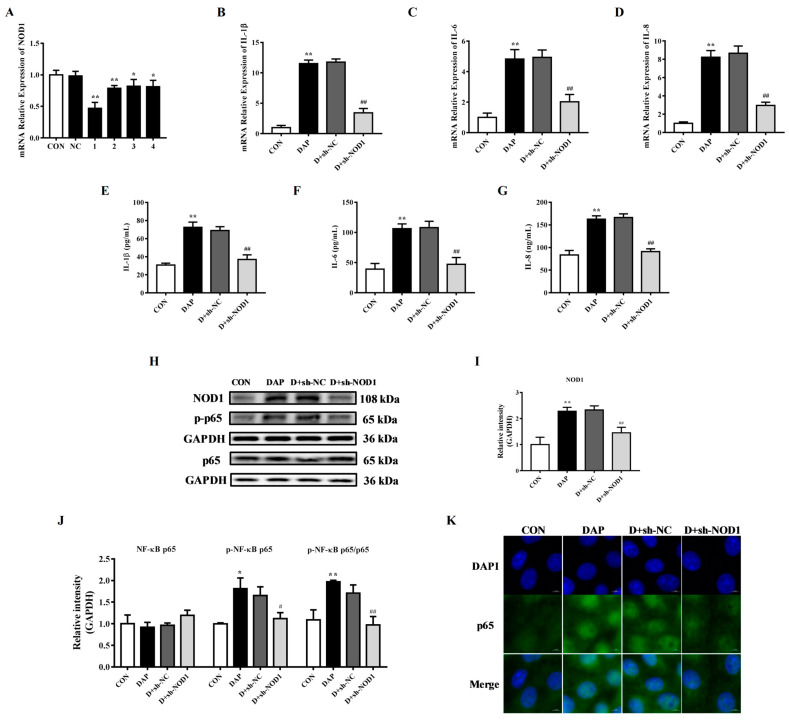
Effects of sh-NOD1 on the inflammatory response induced by iE-DAP in BMECs. (**A**) The gene expression of NOD1 in BEMCs after transfection with NOD1-specific shRNA. (**B**–**D**) Gene expression of inflammatory cytokines in BMECs treated with 1000 ng/mL iE-DAP for 12 h after sh-NOD1 transfection for 36 h. (**E**–**G**) The concentration of inflammatory cytokines in culture supernatant of BMECs treated with iE-DAP after sh-NOD1 transfection. (**H**–**J**) WB representative bands and quantitative results of NOD1/NF-κB signaling pathway-related proteins and (**K**) immunofluorescence results of NF-κB p65 in BMECs treated with iE-DAP after sh-NOD1 transfection. In the immunofluorescence results, the scale bar = 5 μm. Results are reported as the mean ± SEM. * *p* < 0.05, ** *p* < 0.01, represent a significant difference compared with the control group. # *p* < 0.05, ## *p* < 0.01, represent a significant difference compared with the iE-DAP group. D + sh-NC: sh-NC + iE-DAP group; D + sh-NOD1: sh-NOD1 + iE-DAP group.

In order to explore the role of NOD1 on the inflammatory response of BMECs induced by iE-DAP, four NOD1-specific shRNA (shNOD1-1, shNOD1-2, shNOD1-3 and shNOD1-4) were designed and synthesized. After transfecting BMECs, the interference efficiency was tested. As shown in Figure 6A, compared with the control group, shNOD1-1 (*p* < 0.01), shNOD1-2 (*p* < 0.01), shNOD1-3 (*p* < 0.05) and shNOD1-4 (*p* < 0.05) significantly downregulated the relative mRNA expression of NOD1, and the interference efficiency of shNOD1-1 was the highest (Figure 6A).

Figure 6B–G shows that sh-NOD1 transfection hindered the increase in the gene expression and release of the inflammatory cytokines IL-1β (*p* < 0.01), IL-6 (*p* < 0.01) and IL-8 (*p* < 0.01). WB results showed that transfection of sh-NOD1 significantly reduced the protein expression of NOD1 (*p* < 0.01), phosphorylated NF-κB p65 (*p* < 0.05) and the phosphorylation ratio of NF-κB p65 (*p* < 0.01) (Figure 6H–J). Cellular immunofluorescence results further confirmed that NOD1 interference blocked the increased expression of NF-κB p65 and nucleus translocation induced by iE-DAP stimulation (Figure 6K).

### 2.7. Effects of NOD1 Interference on the Tight Junction Disruption in BMECs Induced by iE-DAP

**Figure 7:**
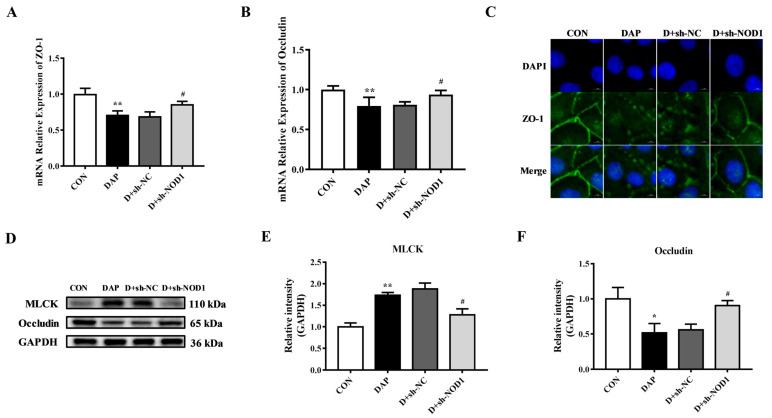
Effects of sh-NOD1 on the tight junction dysfunction induced by iE-DAP in BMECs. (**A**,**B**) Gene expression of ZO-1 and Occludin, (**C**) immunofluorescence results of ZO-1 and (**D**–**F**) WB representative bands and quantitative results of MLCK and Occludin in BMECs treated with 1000 ng/mL iE-DAP for 12 h after sh-NOD1 transfection for 36 h. In the immunofluorescence results, the scale bar = 5 μm. Results are reported as the mean ± SEM. * *p* < 0.05, ** *p* < 0.01, represent a significant difference compared with the control group. # *p* < 0.05 represent a significant difference compared with the iE-DAP group.

After sh-NOD1 transfection, the effects of NOD1 interference on the TJ disruption in BMECs induced by iE-DAP were explored. Figure 7A,B shows that NOD1 interference could reverse the downregulation of the gene expression of ZO-1 (*p* < 0.05) and Occludin (*p* < 0.05) induced by iE-DAP stimulation. WB results also showed that interfering with NOD1 inhibited the decrease in protein expression of Occludin (*p* < 0.01) caused by iE-DAP stimulation (Figure 7D,F). At the same time, immunofluorescence results showed that interference with NOD1 hindered the decreased expression and redistribution of ZO-1 in iE-DAP-stimulated BMECs (Figure 7C). Meanwhile, sh-NOD1 transfection significantly decreased the protein expression of MLCK (*p* < 0.05) compared with the iE-DAP group (Figure 7D,E).

## 3. Discussion

Mastitis is the costliest disease in the dairy industry because it not only impairs production performance but also damages animal health [1,27]. At the same time, mastitis also changes the expression and distribution of TJs, thus damaging the blood-milk barrier and affecting the structure and function of the mammary gland [19]. iE-DAP, a component of the bacterial cell wall, can activate the inflammatory signal pathway and induce an inflammatory response [7,28]. Therefore, our study aims to elucidate the effects of the iE-DAP-induced inflammatory response on TJs in BMECs.

This study has shown that iE-DAP significantly elevated the expression and release of inflammatory cytokines and chemokines in bovine hepatocytes [12]. Consistent with previous studies, this study also found that the mRNA relative expression of inflammatory cytokines IL-1β, IL-6 and IL-8 were significantly upregulated in BMECs after treatment with different concentrations of iE-DAP for 12 h. At the same time, the result of the increased concentration of inflammatory cytokines in the culture supernatant further verified that iE-DAP stimulation could initiate the expression of inflammatory cytokines.

TJ structures in normal mammary epithelial cells can prevent the leakage of milk components during lactation and maintain the normal physiological function of BMECs [29,30]. However, when inflammation occurs, inflammatory cytokines such as IL-1β and IL-6 destroy the integrity of TJs [22,31,32]. It is well known that the structural and functional integrity of TJs is maintained by TJ proteins. Among them, ZO-1 and Occludin play a vital role in the mammary gland [33,34]. This study detected the expression of ZO-1 and Occludin. The results showed that iE-DAP significantly reduced the gene expression of ZO-1 and Occludin in BEMCs. In addition, the integrity of an epithelial barrier is generally evaluated with TEER, and increased leakage of FITC-dextran indicates the permeability of an epithelial barrier [35,36]. Therefore, the decreased TEER and increased paracellular dextran passage in the iE-DAP group indicated that iE-DAP impaired BMEC monolayer integrity. The above results showed that iE-DAP induced an inflammatory response and TJ disruption in BMECs. However, the specific involved mechanism still needed further study.

MLCK played a core role in the process of inflammation-induced increases in intestinal TJ permeability [37]. MLCK could have phosphorylated MLC2, leading to the redistribution of ZO-1 and Occludin and damaging TJ integrity [38]. In order to explore the role of MLCK in iE-DAP-induced TJ disruption, ML-7, an inhibitor of MLCK, was selected for subsequent experiments. The results showed that ML-7 pretreatment significantly reduced the protein expression of phosphorylated MLC2 and the phosphorylation level of MLC2 induced by iE-DAP stimulation and reversed the expression levels and redistribution of TJ proteins in iE-DAP-stimulated BMECs. The barrier function of the BMEC monolayer was also recovered from iE-DAP stimulation with ML-7 pretreatment. It was suggested that the TJ damage induced by iE-DAP stimulation was regulated by the MLCK/p-MLC2 signaling pathway.

NF-κB is an important transcription factor involved in many biological processes including the transcription of inflammatory cytokines [39]. Generally, NF-κB exists in the cytoplasm in an inactive form. Once stimulated by internal and external factors, NF-κB p65 is phosphorylated. Then, phosphorylated NF-κB p65 translocates into the nucleus and promotes gene transcription [40]. At the same time, previous study has shown that LPS treatment could activate the NF-κB signaling pathway and promote the expression of MLCK, thereby increasing the permeability of TJs and damaging the integrity of the intestinal barrier [41]. Vanillin improved the blood-milk barrier function, inhibited the inflammatory response and alleviated the mastitis caused by LPS via inhibiting the activation of the NF-κB signaling pathway [42]. Similar to previous results [43], the current study demonstrated that an NF-κB inhibitor BAY 11-7085 pretreatment reversed the increased phosphorylation ratio of NF-κB and the nucleus translocation of NF-κB p65 in BMECs induced by iE-DAP stimulation. Besides the inhibition of the NF-κB signaling pathway, BAY 11-7085 also significantly reduced the transcription and secretion of inflammatory cytokines stimulated by iE-DAP, which suggested that the NF-κB signaling pathway participated in the regulation of iE-DAP-induced inflammatory responses in BMECs. Excessive cytokine release leads to inflammation-related TJ destruction, while inhibition of cytokine expression could alleviate TJ injury [22,23,24]. In addition to the inhibition of the NF-κB signaling pathway, it was also observed that BAY 11-7085 reversed the alternation in the expression and distribution of TJ proteins as well as TJ dysfunction induced by iE-DAP stimulation in BMECs. At the same time, the upregulation of the protein expression of MLCK in iE-DAP-stimulated BMECs was also inhibited by BAY 11-7085. These results indicated that iE-DAP caused TJ disruption through the NF-κB/MLCK signaling pathway.

As an important member of intracellular PRRs, NOD1 plays an important role in innate immune responses. iE-DAP, as the smallest core structure that NOD1 can recognize [7], could be recognized by the leucine-rich repeat domain of NOD1, which activates NF-κB through a signal cascade reaction and triggers an inflammatory response [10,11]. Interfering with NOD1 in murine macrophages hindered the activation of the downstream signaling pathway in response to iE-DAP treatment [7]. The results of this study showed that iE-DAP significantly increased the gene and protein expression of NOD1. After shRNA interference, the gene and protein expression of NOD1 were significantly decreased, indicating that the synthetic NOD1-specific shRNA could be used to further verify the role of NOD1 in subsequent experiments. Consistent with previous studies [44], sh-NOD1 significantly decreased the phosphorylation ratio of NF-κB and reduced the transcription and secretion of inflammatory cytokines in iE-DAP-treated BMECs. Meanwhile, sh-NOD1 also significantly reduced the protein expression of MLCK and alleviated the TJ disruption. These results suggested that the inflammatory response and TJ disruption in BMECs induced by iE-DAP stimulation were mediated by a NOD1-dependent NF-κB and MLCK signaling pathway.

In conclusion, this study provided evidence that iE-DAP caused an inflammatory response and TJ disruption in BMECs. iE-DAP could be recognized by its specific receptor NOD1, which further activated the downstream NF-κB signaling pathway. Activated NF-κB p65 was translocated into the nucleus to promote the transcription of inflammatory cytokines and induced an inflammatory response. Meanwhile, NF-κB promoted the expression of MLCK, which further activated the MLCK signaling pathway. The increase in inflammatory cytokines and the activation of the MLCK signaling pathway changed the expression and distribution of TJ proteins and damaged TJ barrier function.

## 4. Materials and Methods

### 4.1. Reagents

iE-DAP was brought from InvivoGen (Cat: tlrl-c12dap, San Diego, CA, USA). ML-7 (Cat: HY-15417, purity: 99.63%) and BAY 11-7085 (Cat: HY-10257, purity: 99.99%) were obtained from MedChem Express Inc. (Monmouth Junction, NJ, USA). Reagents employed in cell cultures including RIPM 1640 (Cat: 11875093), 0.25% trypsin (Cat: 25200056), fetal bovine serum (Cat: 10099141) and penicillin-streptomycin solution (Cat: 15140122) were purchased from Gibco (Grand Island, NY, USA). FITC-dextran was brought from Sigma-Aldrich (Cat: FD40, Saint Louis, MO, USA).

### 4.2. Cell Culture

BMECs used in this study were purchased from Shanghai Tongpai Biotechnology Co., Ltd. (Shanghai, China) as previously described [28]. After receiving, the cells were resuscitated and inoculated in the cell resuscitation solution to observe the morphology and growth of the cells. When the cells grew to a confluency of 80% to 90%, they were digested and passaged with trypsin, and some cells were frozen for subsequent experiments. The complete medium for the cell culture was composed with 90% RPMI 1640, 10% fetal bovine serum and 1% penicillin-streptomycin solution. The cells were placed in a cell incubator containing 5% CO_2_ at 37 °C, and the medium was changed for 24~48 h. In this study, 4~9 passage cells were used.

### 4.3. Experimental Design

Firstly, the time gradient test of iE-DAP was carried out with 1000 ng/mL of iE-DAP treatment for different durations (0, 1, 3, 6, 12 and 24 h), and the treatment time was selected as 12 h according to the gene expression of inflammatory cytokines and TJ proteins. Then, samples were collected for subsequent analysis after treatment with different concentration of iE-DAP (0, 1, 10, 100, 1000 and 10,000 ng/mL) for 12 h. Furthermore, different inhibitors and shRNA were used to explore the involved molecular mechanism. BMECs were pre-treated with 10 μM ML-7 (MLCK inhibitor) for 2 h or 10 μM BAY 11-7085 (NF-κB inhibitor) for 1 h, respectively, and then stimulated with 1000 ng/mL of iE-DAP for 12 h. To investigate the role of NOD1 in iE-DAP-induced inflammatory injury, BMECs were transfected with NOD1-specific shRNA for 38 h before iE-DAP treatment.

### 4.4. Cell Viability Assay

An amount of 100 μL of cell suspension containing 5 × 10^3^ BMECs was seeded into a 96-well cell plate and cultured in an incubator at 37 °C and 5% CO_2_ for 24 h. Then, the cells were treated with different concentrations of iE-DAP, ML-7 and BAY 11-7085 for 24 h. The CCK-8 kit was selected to detect the cell viability, and the specific steps were carried out according to the instructions. An amount of 10 μL of CCK-8 solution was added to each well for 2~4 h. Subsequently, the OD value was detected at 450 nm with a microplate reader. During the experiment, the blank control group was set, and 6 replicates were set for each treatment.

### 4.5. Inflammatory Cytokine Concentration Assay

After different cell treatments, the culture supernatant was collected, and the concentration of inflammatory cytokines IL-1β (CK-EN77024), IL-6 (CK-EN77030) and IL-8 (CK-EN77031) in culture supernatant were detected using a bovine ELISA kit (Nanjing Hongsheng Biotechnology Co., Ltd., Nanjing, China) according to the manufacturer’s instructions.

### 4.6. Real-Time Quantitative PCR Assay

The cells were seeded into 12-well cell plates, and after corresponding cell treatment, the culture medium was discarded. The cells were washed with 1 × PBS and then incubated with 500 μL of RNA extraction reagent RNAiso plus (Cat: 9109, Takara Co., Otsu, Japan) for 3 min. The cell lysate was collected into an RNAase-free EP tube and vortexed for 10 s. Subsequent steps were conducted as previously described [45]. Briefly, the quality and concentration of total RNA was assessed with NanoDrop 2000 (Eppendorf Biotechnology, Hamburg, Germany) and agarose gel electrophoresis after RNA extraction. cDNA reverse transcription was performed with the Hifair II 1st Strand cDNA Synthesis SuperMix (Cat: 11120ES60, Yeasen Biotechnology Co., Ltd., Shanghai, China). Subsequently, real-time quantitative PCR (RT-qPCR) assay was conducted using ChamQ Universal SYBR qPCR Master Mix (Cat: Q711, Vazyme Biotech Co., Ltd., Nanjing, China). The primers used in this experiment are listed in Supplemental Table S1 and GAPDH was selected as an internal control. The fold change in mRNA expression related to GAPDH was calculated using the 2^−ΔΔCt^ method.

### 4.7. Western Blotting Assay

The cells were seeded into 6-well cell plates, and after corresponding cell treatment, the culture medium was discarded. Then the cells were washed with 1× PBS and lysed with 150 μL of protein extraction reagent RIPA lysis buffer (Cat: BL504A, Biosharp Life Sciences, Hefei, China) adding protease inhibitor PMSF for 3 min. The cell lysate was collected into an EP tube with a cell scraper and then centrifugated at 12,000 rpm/min for 5 min at 4 °C to extract total cell protein. Subsequent steps were conducted as previously described [45]. Briefly, cell protein concentration was detected using a BCA Protein Assay kit (Cat: 23225, ThermoFisher Scientific, Waltham, MA, USA). Next, equal amounts of denatured protein were separated with sodium dodecyl sulfate polyacrylamide gel electrophoresis and then transferred onto polyvinylidene fluoride membranes (Bio-Rad Life Science Research, Hercules, CA, USA). The transferred membranes were incubated with specific primary antibodies. GAPDH was employed as a reference protein. After washing, the membranes were further incubated with corresponding secondary antibodies. The specific information of antibodies is listed in Supplemental Table S2. The results were visualized using a ChemiDoc MP system (Bio-Rad) and analyzed with Bio-Rad Image Lab 5.2.1 Software (Bio-Rad).

### 4.8. Transepithelial Electrical Resistance and Paracellular Permeability Assay

The cells were plated and grown as monolayers on a 12-well cell culture-chamber-insert system (0.4 μm, Labgic Company, Beijing, China). After corresponding cell treatment, the transepithelial electrical resistance (TEER) was detected with a Millicell ERS-2 Epithelial Volt-Ohm Meter (Merck Millipore, Burlington, MA, USA) in accordance with the instructions. The results of the TEER were expressed as a percentage relative to the control group.

To detect paracellular permeability, FITC-dextran was added to the upper chamber and incubated for 4 h. Then, samples were collected from the bottom chamber to assess the fluorescence intensity with a fluorescence spectrometer (F-7000, Hitachi, Japan) following the instructions. A standard curve was built to calculate the concentration of FITC-dextran and the percentage relative to the control group.

### 4.9. Cellular Immunofluorescence Assay

BMECs were seeded into 12-well cell plates which were equipped with sterile cell coverslips. After corresponding cell treatment, cellular immunofluorescence assay was performed as described next. The cells were fixed with 4% paraformaldehyde for 20 min after 1 × PBS wash and then incubated with 0.3% Triton X-100 for 15 min to increase cellular membrane permeability. Then the cells were further blocked with blocking solution containing 5% bovine serum albumin for 30 min followed by incubation overnight at 4 °C with the specific primary antibody. After washing, the coverslips were incubated for 60 min at 37 °C with the corresponding FITC-linked secondary antibody. Antibodies used in the cellular immunofluorescence assay are also summarized in the Supplemental Table S2. Then, the results were visualized using an LSM 710 confocal laser microscope system (Zeiss, Oberkochen, Germany) after the cell nuclear staining with DAPI solution (Cat: C0060, Solarbio Life Sciences, Beijing, China). This was kept gently operating throughout the whole experiment. The experimental conditions, especially the permeabilization time, could be optimized according to the characteristics of the different target proteins.

### 4.10. Short Hairpin RNA Transfection Assay

NOD1-specific shRNA was designed and synthesized by a commercial company (Tsingke Biotechnology, Beijing, China) according to the mRNA sequence of NOD1 (NM_001256563). Plasmid transformation and bacteria amplification were performed following the product protocols. Subsequently, plasmids were extracted using endotoxin-free plasmid extraction reagent (Tsingke Biotechnology). When BEMCs reached 70~90% confluency, transfection reagent Lipofectamine 3000 (ThermoFisher Scientific) was selected to carry out cell transfection according to the reagent instructions. After incubation, samples were collected for further analysis.

### 4.11. Data Statistical Assay

The results were analyzed by a one-way ANOVA using the statistical software IBM SPSS 20.0 (IBM Inc., Armonk, NY, USA), and a Dunnett’s test was used for multiple comparisons. The normality of the distribution of the variables was analyzed before statistical assay. Each test was independently repeated 3 times and had 3 replicates. All data were expressed as the mean ± the standard error of the mean (mean ± SEM). When significance was *p* < 0.05, it was considered significantly different. GraphPad Prism 7 (GraphPad Software, La Jolla, CA, USA) and EDraw Max software (EDraw Max Inc., shenhzen, China) were employed to create all the figures.

## Data Availability

The data that support the findings of this study are available from the corresponding author upon reasonable request.

## Supplementary Materials

The supporting information can be downloaded at: https://www.mdpi.com/article/10.3390/ijms24076263/s1.
