# Supplementary material for: iE-DAP Induced Inflammatory Response and Tight Junction Disruption in Bovine Mammary Epithelial Cells via NOD1-Dependent NF-κB and MLCK Signaling Pathway
Source: Int J Mol Sci. 2023 Mar 27;24(7):6263. doi: 10.3390/ijms24076263 (PMC10094069; doi:10.3390/ijms24076263)
Supplement: Supplementary file 1 [file ijms-24-06263-s001.zip › ijms-2288492-supplementary.pdf]

Supplementary materials

Table S1. Primer sequences used for real-time quantitative PCR

Gene	Forward primer (5'-3')	Reverse primer (5'-3')	NCBI accession	length(bp)
IL-1β	CTATTCTCTCCAGCCAACCTTC	CTCGTCACTGTAGTAAGCCATC	NM_174093.1	100
IL-6	GGAGGAAAAGGACGGATGCT	GGTCAGTGTTTGTGGCTGGA	NM_173923.2	227
IL-8	CCTCTTGTTCAATATGACTTCCA	GGCCCACTCTCAATAACTCTC	NM_173925.2	189
ZO-1	GCGAAATGAGAAACAAGCACC	ATGAGTTGAGTTGGGCAGGAC	XM_024982012.1	121
Occludin	CAGCAGCAGTGGTAACTTGGA	CCGGTCGTGTAGTCTGTTTCAT	NM_001082433.2	111
GAPDH	GGGTCATCATCTCTGCACCT	GGTCATAAGTCCCTCCACGA	NM_001034034.2	176

Table S2. Information of the antibodies used in western blotting analysis and cellular immunofluorescence

Target protein	Catalog number	Application	Company
NOD1	SC22045	Primary antibody	Santa Cruz Biotechnology
NF-κB p65	AF1234	Primary antibody	Beyotime Biotechnology
phosphorylated NF-κB p65	AN371	Primary antibody	Affinity Biosciences LTD
ZO-1	21773-1	Primary antibody	Proteintech Group
Occludin	ab167161	Primary antibody	Abcam Plc
MLCK	AF1678	Primary antibody	Beyotime Biotechnology
MLC2	DF7911	Primary antibody	Affinity Biosciences LTD
phosphorylated MLC2	AF5443	Primary antibody	Affinity Biosciences LTD
GAPDH	10494-1	Primary antibody	Proteintech Group
Anti-rabbit HRP-linked Antibody	A0208	Secondary antibody	Beyotime Biotechnology
Anti-mouse HRP-linked Antibody	A0216	Secondary antibody	Beyotime Biotechnology
Anti-goat HRP-linked Antibody	S0010	Secondary antibody	Affinity Biosciences LTD
Anti-rabbit FITC-linked Antibody	A0208	Secondary antibody	Beyotime Biotechnology
Anti-mouse FITC-linked Antibody	A0216	Secondary antibody	Beyotime Biotechnology
